# Investigation of factors influencing intravenous glucocorticoid treatment course in severe acute urticaria: A retrospective study

**DOI:** 10.1097/MD.0000000000049628

**Published:** 2026-07-03

**Authors:** Kun Wang, Mingyi Liao, Shiyin Xiong, Xinhao Wu, Zhaolin Zeng, Cong You

**Affiliations:** a Department of Dermatology and Venereology, Candidate Branch of National Clinical Research Centre for Skin and Immune Diseases, The First Affiliated Hospital of Gannan Medical University, Ganzhou, People’s Republic of China; b Department of Dermatology, Ganzhou People’s Hospital, Ganzhou, Jiangxi, China; c Health Science Center, Jinggangshan University, Ji’an, Jiangxi, China.

**Keywords:** acute urticaria, infection, intravenous glucocorticoids, neutrophil

## Abstract

Acute urticaria, a prevalent skin condition characterized by wheals and itching, often requires intravenous glucocorticoids for patients with severe symptoms and systemic manifestations. However, clinical studies investigating factors influencing the intravenous glucocorticoid treatment course in patients with severe acute urticaria are lacking. This retrospective study examines factors influencing intravenous corticosteroid treatment in severe acute urticaria and compares the characteristics of patients with and without infection. This study enrolled 96 patients with severe acute urticaria. Baseline data – sex, age, region, season, accompanying symptoms, triggers, glucocorticoid types, initial dose, neutrophil, platelet, lymphocyte, white blood cell counts, Urticaria Activity Score, and treatment duration were meticulously recorded for each participant. Spearman correlation, Mann–Whitney *U* and Kruskal–Wallis *H* tests explored relationships between the treatment course of intravenous glucocorticoids and continuous or categorical variables. Statistically significant variables identified in the univariate analysis were tested using multiple linear regression analysis. Chi-square tests or Spearman correlation were used to compare differences between the infection-triggered and non-infection-triggered groups. Multiple linear regression analysis revealed infection (β = 0.05, [95% confidence interval: 0.02–2.09], *P* < .05) and elevated neutrophils (β = 1.47, [95% confidence interval: 0.09–2.85], *P* < .05) to be significantly associated with a prolonged duration of intravenous glucocorticoid therapy in severe acute urticaria patients. No significant differences were observed between infectious and noninfectious acute urticaria groups in the subgroup analysis. Infection and elevated neutrophil count were associated with prolonged intravenous glucocorticoid courses. Investigating other laboratory indicators associated with infection-triggered severe acute urticaria, rather than the neutrophil, lymphocyte, and white blood cell levels, is recommended.

## 1. Introduction

Acute urticaria, a prevalent skin condition, is characterized by the sudden appearance of wheals, angioedema, or both, typically resolving within 6 weeks.^[1]^ During this phase, immunoglobulin E (IgE) antibodies attach to specific receptors on mast cells and basophils. This triggers the release of inflammatory substances, including histamine, platelet-activating factors, and cytokines, which are responsible for the development of wheals and angioedema.^[2]^ Research findings indicate that a significant proportion of individuals (approximately 12–20%) will develop at least one subtype of urticaria during their lifetime.^[3]^ In a Chinese national survey, the lifetime prevalence of urticaria in the population was 7.3%, slightly higher in women (8.92%) than men (6.61%).^[4]^ Among urticaria subtypes, acute urticaria is the most common form.^[4]^ Common triggers encompass infections, acute allergic reactions to drugs, foods, or insect bites.^[3,5]^ Several studies have identified respiratory and other microbial infections as frequent triggers.^[6]^ Acute urticaria can significantly disrupt sleep, diminish quality of life, and impose psychological burdens. Research indicates a noteworthy probability, ranging from 20 to 45%, of acute urticaria progressing into chronic urticaria, with symptoms lasting over 6 weeks.^[7]^ Patients with chronic urticaria endure prolonged periods of rash and itching, along with substantial psychological and economic burdens. Therefore, timely and appropriate therapy is needed to minimize the risk of acute urticaria progressing into a chronic state, thereby improving patients’ prognosis and quality of life. Severe acute urticaria is often accompanied by respiratory or digestive symptoms such as abdominal pain, diarrhea, nausea, vomiting, dyspnea, and dysphagia. These patients frequently require hospitalization.^[8–10]^ According to the guidelines for the diagnosis and treatment of urticaria, systemic glucocorticoids may be considered alongside second-generation nonsedative antihistamines for severe symptoms.^[1,10]^ However, intravenous glucocorticoid duration should be minimized to reduce potential adverse effects like high blood pressure, hyperglycemia, and peptic ulcers. The Urticaria Activity Score (UAS) is a widely used tool for evaluating urticaria activity. It is an essential indicator for assessing severity, monitoring treatment effectiveness, and guiding treatment planning.^[11,12]^ However, clinical studies investigating factors influencing the intravenous glucocorticoid treatment course in patients with severe acute urticaria are lacking. In this study, we aimed to explore the clinical characteristics of patients with severe acute urticaria and identify factors affecting the treatment duration of intravenous glucocorticoids at our hospital. In addition, we compared the characteristics of patients with severe acute urticaria caused by infection with those who were not affected.

## 2. Materials and methods

This study is based on a retrospective analysis of clinical data from patients with severe acute urticaria hospitalized in the Department of Dermatology at the First Affiliated Hospital of Gannan Medical University from March 2023 to January 2024. This study adhered to the principles of the Declaration of Helsinki of the World Medical Association and ethics approval was granted by the Ethics Committee of the First Affiliated Hospital of Gannan Medical University (No. LLSC-2023021307). All the patients provided written informed consent for the publication of their data. The study entailed searching electronic databases using the International Classification of Diseases, tenth edition code L50.800*003 (severe acute urticaria) to pre-identify eligible patients. According to the guidelines, the diagnostic criteria for severe acute urticaria were as follows: “spontaneous pruritic wheals throughout the body, which resolve spontaneously without leaving traces, accompanied by respiratory and digestive symptoms, such as dyspnea, abdominal pain, and diarrhea, etc.”^[10,13]^ Intravenous glucocorticoids are indicated for acute urticaria when there are severe disseminated wheals and systemic respiratory and digestive symptoms.^[5,10,14,15]^ All physicians made diagnostic assessments following uniform criteria. Retrospective verification of International Classification of Diseases, tenth edition code L50.800*003 was conducted to ensure accurate case classification.

In this study, patient triggers were classified as infection if the levels of immunoglobulin M of influenza virus, parainfluenza virus, and antistreptolysin O were elevated or if bacterial cultures of clinical samples tested positive or if chest X-rays indicated pulmonary infection.^[13,16]^ The exclusion criteria were as follows: pregnant women, individuals with serious systemic diseases like severe cardiovascular disorders or anaphylactic shock, those on immunosuppressants or contraindicated for glucocorticoid application, patients with cancer, patients who underwent midway transfer or automatic discharge, or those with incomplete personal information, among others.

**Figure 1.**
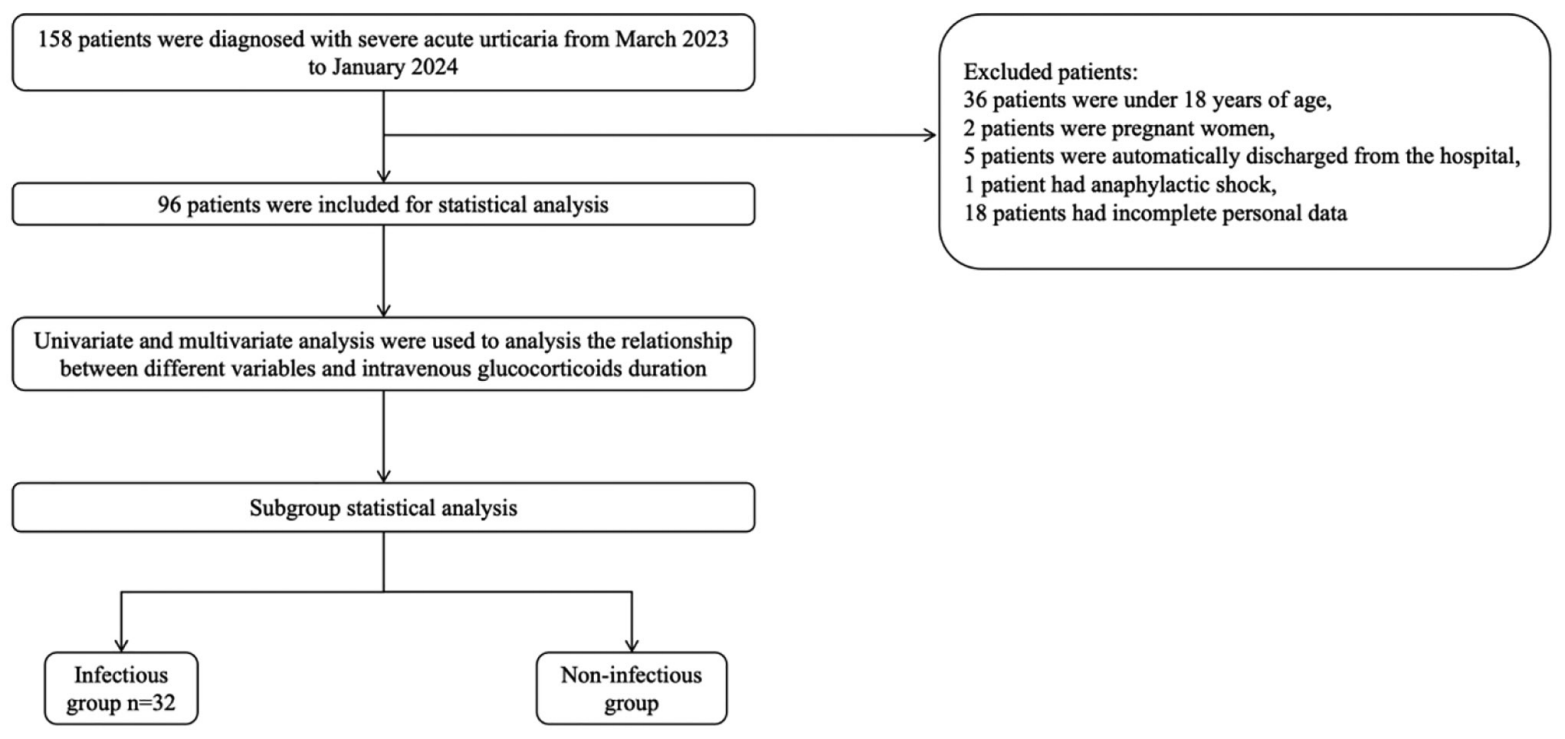
Study flowchart of materials and methods.

A total of 158 patients were initially identified, with 62 patients excluded. These exclusions included 36 patients under 18, 2 pregnant women, 5 automatically discharged patients, 1 with anaphylactic shock, and 18 with incomplete personal data. Ultimately, 96 patients were included in the analysis (Fig. 1). Baseline patient characteristics were collected, including sex; age; region; season; concomitant symptoms; triggers; types of intravenous glucocorticoids used for treatment; initial dose of glucocorticoids; levels of neutrophils, platelets, lymphocytes, and white blood cells (WBCs); UAS at admission; and treatment duration. The UAS comprises 2 components: the count of urticarial wheals and the degree of itching experienced by the patient. The overall score ranges from 0 to 6, with higher scores indicating more severe urticaria activity.^[11,17]^ The baseline characteristics of the patients with acute urticaria are shown in Table 1.

**Table 1:** Baseline characteristics of patients with severe acute urticaria (n = 96).

Characteristics	Cases n (%)/IQR
Gender	
Male	26 (27.08)
Female	70 (72.92)
Age (yrs)	(30.25–49.75)
Area	
Rural	64 (66.67)
Urban	32 (33.33)
Season	
Spring	25 (26.04)
Summer	35 (36.46)
Autumn	23 (23.96)
Winter	13 (13.54)
Concomitant symptoms	
Respiratory symptoms	39 (40.63)
Digestive symptoms	40 (41.67)
Both	17 (17.70)
Triggers	
Infection	32 (33.33)
Non-infection	64 (66.67)
Types of glucocorticoids used for treatment	
Dexamethasone	62 (64.58)
Methylprednisolone	34 (35.42)
Initial dose of glucocorticoids (prednisone [mg/kg·d])	(0.74–1.11)
The level of neutrophils	
Normal	82 (85.42)
Elevated	14 (14.58)
The level of platelets	
Normal	69 (71.88)
Elevated	27 (28.12)
The level of lymphocytes	
Normal	85 (88.54)
Reduced	11 (11.46)
The level of WBC	
Normal	14 (14.58)
Elevated	82 (84.42)
UAS on admission	(4–5)
Duration of intravenous glucocorticoids (d)	(4–6)

The normal range of number of neutrophils is 1.8–6.3 × 10^9^/L; the normal range of number of platelets is 125–350 × 10^9^/L; the normal range of number of lymphocytes is 0.8–4.0 × 10^9^/L; and the normal range of numbers of WBC is 3.5–4.5 × 10^9^/L.

IQR = interquartile range, UAS = Urticaria Activity Score, WBC = white blood cell.

### 2.1. Laboratory tests and treatment regimens

As observed in the electronic medical record database, blood cell analyses were conducted on 96 patients. Two types of corticosteroids were identified in the prescriptions: methylprednisolone (Huabang Pharmaceutical Co. Ltd.) and dexamethasone (Suicheng Pharmaceutical Co., Ltd.). According to the medical records, all the patients recovered fully. In this study, discontinuing intravenous corticosteroid treatment was determined based on the resolution of wheals and the absence of systemic symptoms within 24 hours, leading to a UAS score of 0. The relevant judgment criteria were summarized retrospectively from archived medical records.

### 2.2. Statistical analysis

SPSS V26.0 (IBM Corp) was used for statistical analysis. Categorical variables were expressed as frequencies, and continuous variables as interquartile ranges. The Mann–Whitney *U* test or Kruskal–Wallis H test was used to compare the differences in the duration of intravenous glucocorticoid treatment across categorized variables. Simple linear analysis was performed using Spearman rank analysis for continuous variables. In cases where variables significantly differed in univariate analysis, multiple linear regression was applied for multivariate analysis. The chi-square test or Mann–Whitney *U* test was used to compare differences between variables in the infectious and noninfectious groups. Statistical significance was set at *P* < .05. GraphPad Prism 6.0 (GraphPad Software) was used for data visualization.

## 3. Results

From March 2023 to January 2024, 96 patients (26 males and 70 females) were enrolled in this retrospective study. Of these, 66.67% were from rural areas, with the primary onset seasons of acute urticaria being spring and summer (62.5%). A total of 32 patients were considered to be triggered by infection. Among the infected patients, 9 tested positive for antistreptolysin O antibodies, 5 for parainfluenza virus antibodies, and 7 patients for influenza virus antibodies. Additionally, 3 patients had chest radiographs showing lung infections, and 8 showed positive secretion bacterial cultures. Noninfectious triggers were identified in 64 patients: 6 were food-induced, 2 were triggered by inhalants, and triggers were not found for the rest. All patients displayed varying degrees of systemic symptoms; precisely, 39 presented with respiratory symptoms, 40 with digestive symptoms, and 17 with a combination of both. All 96 patients underwent blood cell analyses, revealing elevated neutrophils in 14 cases, elevated platelet counts in 27 cases, elevated WBC counts in 82 cases, and reduced lymphocyte counts in 11 cases.

**Figure 2.**
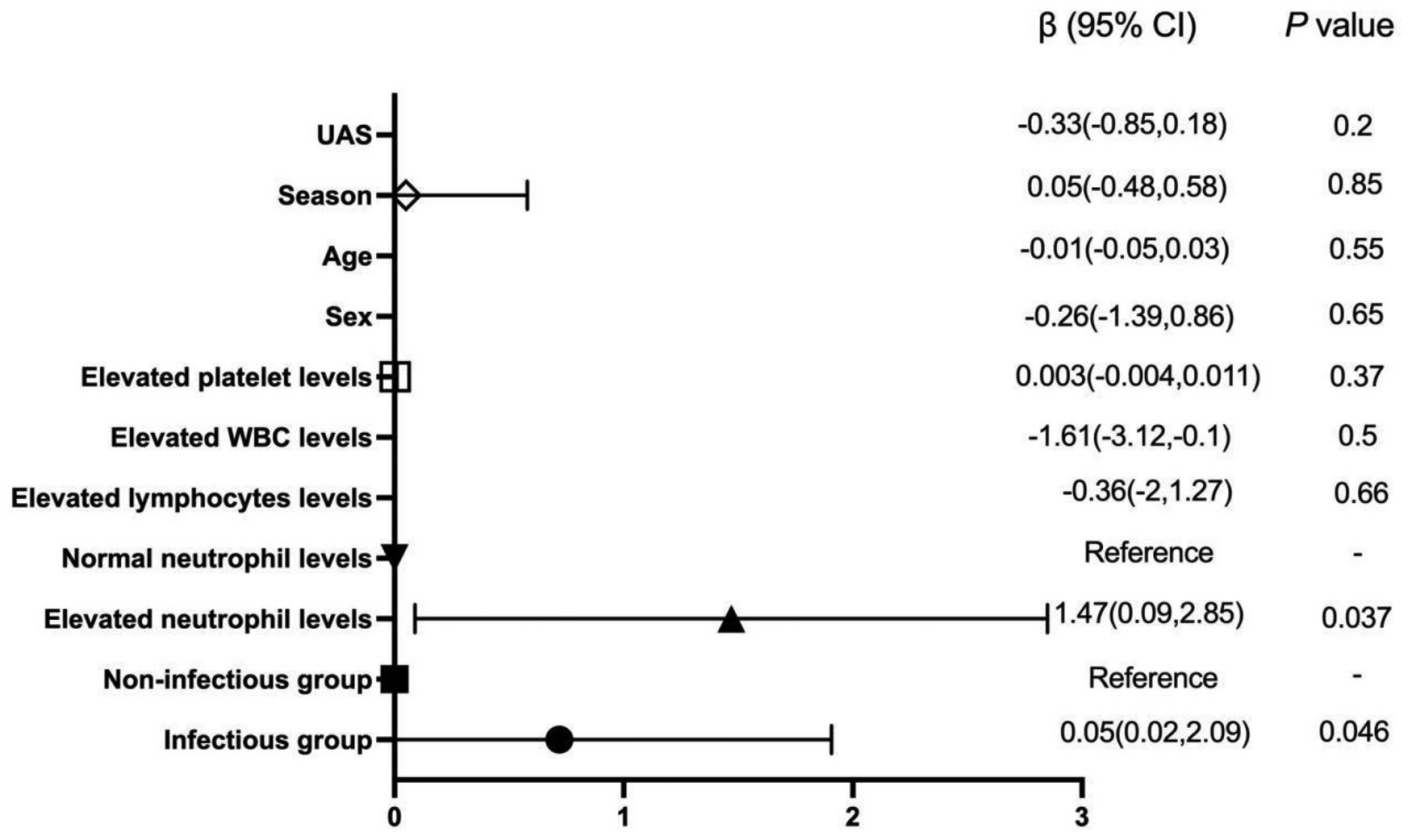
Multiple linear regression analysis of factors affecting the duration of intravenous glucocorticoids for severe acute urticaria. Note: The horizontal axis is presented on a logarithmic scale. The 95% confidence interval (0.02–2.09) for infection was calculated on the linear scale. Since the interval does not contain zero, the result was statistically significant (*P* < .05). The visual crossing of zero on the logarithmic axis is due to scale transformation. CI = confidence interval, UAS = Urticaria Activity Score.

Univariate analysis showed positive associations between neutrophil counts or infections with the duration of intravenous glucocorticoid administration in patients with acute urticaria (Table 2, *P* < .05). Multiple linear regression analysis indicated that infection (Fig. 2, β = 0.05, [95% confidence interval [CI]: 0.02–2.09], *P* < .05) and elevated neutrophil count (Fig. 2, β = 1.47, [95% CI: 0.09–2.85], *P* < .05) were correlated with prolonged duration of intravenous glucocorticoid therapy. Due to wide and asymmetric CIs, the findings should be interpreted with caution. Conversely, no statistically significant associations were observed between variables such as sex, age, region, season, accompanying symptoms, glucocorticoid type, and starting dose, platelet level, lymphocyte level, WBC level, UAS, and the length of intravenous glucocorticoid therapy (Tables 2 and 3). We observed no statistically significant differences in sex, age, area, season, accompanying symptoms, neutrophils, platelets, lymphocytes, WBC, and UAS levels between the infectious and noninfectious groups (Table 4).

**Table 2:** Single-factor analysis of categorized variables affecting the treatment duration of intravenous glucocorticoids in patients with severe acute urticaria.

Variables	Group	Treatment duration (days, IQR)	*Z*/*H*	*P* *
Gender	Male	(4, 6)	–0.74	.46
	Female	(3, 6)		
Area	Rural	(4, 6)	0.37	.71
	Urban	(4, 6)		
Season	Spring	(4, 6)	3.00	.39
	Summer	(3, 6)		
	Autumn	(4, 6)		
	Winter	(4.5, 7.5)		
Concomitant symptoms	Respiratory system	(3, 6)	2.23	.33
	Digestive system	(4, 7)		
	Both	(3.50, 6)		
Triggers	Infection	(4, 7)	–2.12	.03
	Non-infection	(3, 6)		
Glucocorticoids used for treatment	Dexamethasone	(4, 6)	–0.92	.36
	Methylprednisolone	(3, 6.25)		
The level of neutrophils	Normal	(3.75, 6)	–2.19	.03
	Elevated	(5, 8.5)		
The level of platelets	Normal	(4, 6)	–0.07	.94
	Elevated	(4, 6)		
The level of lymphocytes	Normal	(4, 6)	–0.20	.84
	Reduced	(4, 7)		
The level of WBC	Normal	(4.75, 7.75)	–1.85	.07
	Elevated	(3, 6)		

IQR = interquartile range, WBC = white blood cell.

* *P* values were calculated using the Mann–Whitney *U* test or Kruskal–Wallis test.

**Table 3:** Spearman rank correlation tests of continuous variables affecting the duration of intravenous glucocorticoids in patients with severe acute urticaria.

Variables	*r*	*P*
Age (yrs)	–0.06	.58
The initial dose of glucocorticoids [prednisone (mg/kg·d)]	–0.11	.30
UAS on admission	0.001	1.0

UAS = Urticaria Activity Score.

**Table 4:** Comparative analysis between infectious and noninfectious patient groups with severe acute urticaria.

Characteristics	Group	Infectious (n/IQR)	Noninfectious (n/IQR)	χ^2^/*Z*	*P* *
Gender	Male	8	18	0.11	.75
	Female	24	46		
Age(years)	–	(30.75–47.5)	(30.25–51)	–0.53	.60
Area	Rural	20	44	0.38	.54
	Urban	12	20		
Season	Spring	9	16	1.49	.69
	Summer	10	25		
	Autumn	7	16		
	Winter	6	7		
Concomitant symptoms	Respiratory system	10	29	3.99	.14
	Digestive system	13	27		
	Both	9	8		
The level of neutrophils	Normal	29	53	1.05	.30
	Elevated	3	11		
The level of platelets	Normal	22	47	0.23	.63
	Elevated	10	17		
The level of lymphocytes	Normal	27	58	0.82	.37
	Reduced	5	6		
The level of WBC	Normal	3	11	1.05	.30
	Elevated	29	53		
UAS	–	(4–5.75)	(4–5)	–1.75	.08

IQR = interquartile range, UAS = Urticaria Activity Score, WBC = white blood cell.

* *P* value were calculated using the chi-square test or Mann–Whitney *U* test.

## 4. Discussion

In most cases, acute urticaria is associated with a positive prognosis. However, it is important to note that individuals with severe acute urticaria are at a higher risk of developing laryngeal edema and experiencing breathing difficulties. In such cases, hospitalization and the administration of systemic corticosteroids are often recommended to manage the condition effectively and prevent potential complications.^[14]^ Previous studies have reported a higher incidence of acute urticaria in women and among young to middle-aged patients, consistent with our current findings.^[18–20]^ Similar to previous studies, our results showed that the main onset season for acute urticaria is concentrated in spring and summer (53.85%),^[21,22]^ potentially linked to the high incidence of mosquito bites and pollen from plants during these seasons. In our study, most patients were from rural areas (65.4%), which is inconsistent with the results of Jadhav and Silvares et al, who found that urban residents had a higher incidence of allergic diseases. This discrepancy may be attributed to urban residents’ increased exposure to allergenic pollutants from factories and vehicles as opposed to their rural counterparts.^[20,23]^

Acute urticaria is generally more likely to have an identifiable cause than chronic urticaria. Nonetheless, it is important to note that even in cases of acute urticaria, approximately 30 to 50% of cases are still considered idiopathic, meaning that the underlying cause remains unknown.^[24–26]^ Similar to the study by Kulthanan et al, more than half of the adult patients in our study had no identifiable etiology.^[13]^ Identifying the trigger is crucial in the treatment of acute urticaria as it plays a significant role in determining the duration and effectiveness of therapeutic interventions. In most cases where a specific trigger can be identified, infection stands out as a common underlying cause. For example, a study by Aoki et al found that 64% of patients exhibited symptoms indicative of acute bacterial or viral infection as a possible trigger for urticaria.^[24]^

The UAS; is a score that combines the number of wheals per day with the degree of pruritus.^[12,27]^ It is a commonly employed tool for evaluating urticaria’s severity and treatment efficacy. However, our study revealed no statistically significant correlation between the UAS score and the duration of intravenous glucocorticoid treatment for severe acute urticaria. Therefore, we infer that the UAS may have limitations gauging the severity of acute urticaria compared to chronic urticaria.^[28]^ First, the UAS primarily emphasizes the degree of itching and number of wheals while potentially overlooking other crucial factors. Severe acute urticaria is commonly associated with a spectrum of systemic manifestations, including laryngeal edema, dyspnea, hemodynamic instability, abdominal pain, and diarrhea. As the UAS fails to comprehensively reflect these systemic features, its capacity for the holistic assessment of acute urticaria is substantially limited. Accordingly, even in patients with relatively mild cutaneous symptoms, severe systemic inflammation and high-risk conditions may already be present, which markedly compromises the clinical applicability of the UAS in the evaluation of severe acute urticaria. Second, acute urticaria tends to develop and fluctuate rapidly over a short period, making it difficult to accurately assess the severity of the patient’s condition during UAS evaluation, as they might not be in their most severe state. Second, acute urticaria tends to develop and fluctuate rapidly over a short period, making it difficult to accurately assess the severity of the patient’s condition during UAS evaluation, as they might not be in their most severe state.

In our study, 32 patients were considered to be triggered by infection. The relationship between acute urticaria and infection has gained significant attention, with numerous studies reporting an association between bacterial and viral infections and the onset of acute urticaria. Specific pathogens such as streptococci, hepatitis A and B viruses, and influenza A and parainfluenza viruses have been highlighted as potential triggers of acute urticaria.^[29–31]^ Zhang et al found detectable antistreptococcal antibodies in many adults with asymptomatic acute idiopathic urticaria, indicating that latent infections may trigger urticarial hypersensitivity reactions, yet the specific mechanisms remain unclear.^[32]^ Viral IgM and IgG antibodies can cross-react with mast cell IgE to promote mast cell degranulation, while Malassezia extracts have been proven to potentiate IgE-dependent mast cell activation and amplify allergic inflammation.^[33,34]^ In general, bacterial and viral infections drive urticaria exacerbation through multiple pathways: they rapidly induce mast cell and basophil degranulation and histamine release via antigen-antibody complexes, causing vascular hyperpermeability; they also activate antigen-presenting cells and T cell-related inflammatory cascades, further aggravating urticaria symptoms.^[35–41]^ Our study found that patients with infection-associated urticaria require long-term intravenous glucocorticoidtherapy. This result suggests that infection not only exacerbates the patient’s condition but may also impact the therapeutic effect of glucocorticoids. As a common anti-inflammatory drug, glucocorticoids exert their anti-inflammatory effects primarily by binding to glucocorticoid receptors (GR) in the cytoplasm.^[42]^ This binding dissociates the GR complex from its sequestration in the target cell, leading to GR activation and rapid translocation into the nucleus.^[42]^ Multiple studies have demonstrated that infections with bacteria, *Mycoplasma pneumoniae*, and respiratory syncytial virus reduce GR nuclear translocation, resulting in glucocorticoid insensitivity and an increased risk of drug resistance.^[43–45]^ Overall, the aforementioned studies indicate that the exacerbation of severe acute urticaria by infection is the result of multiple mechanisms working together.^[46]^ The core lies in infection-induced activation of the inflammatory response, immune system, and mast cells, leading to increased release of inflammatory factors. Simultaneously, infection leads to glucocorticoid insensitivity, prolonging the treatment course and forming a bidirectionally regulated scenario of “pathophysiological disorders – treatment resistance.”

Neutrophils are the most abundant type of circulating WBCs and are the initial defense against bacterial and viral infections. In dermatological practice, cases of acute urticaria accompanied by elevated leukocyte, lymphocyte, or neutrophil levels are frequently encountered, and such changes in blood cell counts often raise suspicion of infection as an underlying cause. However, our study revealed that leukocyte, lymphocyte, and neutrophil counts in patients with acute urticaria did not differ significantly between those with and without infection. This suggests that these 3-blood cell counts alone may not reliably indicate the presence of infection in patients with severe acute urticaria. To identify more reliable and sensitive indicators of infection-triggered acute urticaria, exploring the potential association between other biomarkers; inflammatory mediators, such as specific cytokines, chemokines, and immune cell profiles, is imperative. Additionally, our study revealed that elevated WBC and lymphocyte counts were not significantly associated with the course of intravenous glucocorticoid treatment, indicating that they did not reflect the severity of the allergy. Lymphocytes and leukocytes are diverse groups of cells consisting of multiple subsets and types, each with distinct functions in the context of anaphylaxis. Thus, subgroups of lymphocytes and leukocytes may offer a more comprehensive reflection of the severity of allergic reactions rather than the counts themselves.

In contrast to lymphocyte and leukocyte counts, this study found that elevated neutrophils were positively correlated with the treatment duration of severe acute urticaria. Neutrophils are produced in the bone marrow. During an allergic reaction, neutrophils are the first cells to reach inflammatory sites, entering the circulation and accumulating at inflammatory loci within approximately 1 hour, which leads to an increase in circulating neutrophils.^[47]^ Previous studies regarded neutrophilia as a secondary manifestation of inflammation, suggesting that it only represents a defensive response of the body to inflammatory stimuli – a compensatory defense mechanism initiated after disease onset. However, numerous recent studies support the view that neutrophils are also involved in the pathogenic process.^[48–50]^ To date, the association between neutrophils and allergic diseases has been extensively documented. Neutrophils are essential for inflammation and allergen-inspired T-cell recruitment, highlighting their role in the inflammatory induction phase of allergic skin conditions.^[51]^ Numerous studies have reported elevated neutrophil-to-lymphocyte ratios in allergic diseases including allergic rhinitis and atopic dermatitis. This ratio is largely driven by marked changes in neutrophil counts and may be closely correlated with disease severity.^[52–54]^ Recent methodological advances in pregnostic modeling of inflammatory conditions have similarly identified neutrophil-based metrics as worth predictors of clinical outcomes.^[55,56]^ The pathogenic role of neutrophils is closely linked to neutrophil extracellular traps (NETs). Neutrophils release NETs, which exacerbate inflammatory tissue injury and may contribute to increased systemic autoantibody production.^[57]^ Specifically, an increased neutrophil count provides a sufficient cellular basis for NET formation. In acute urticaria, rising neutrophil counts are accompanied by a greater number of activated neutrophils, significantly increasing the likelihood of NET formation. NET formation, in turn, further amplifies the inflammatory response and promotes tissue damage. Meanwhile, interleukin-6 (IL-6), a key proinflammatory cytokine, has been shown to be significantly elevated in severe acute urticaria.^[58]^ This indicates that IL-6 is closely associated with disease severity and may serve as a potential biomarker for assessing disease severity. Neutrophils are considered one of major sources of IL-6, a multigenic cytokine involved in the inflammatory response, correlates with the severity or pathogenesis of allergic diseases, including asthma, urticaria, and mastocytosis.^[59–61]^ On the other hand, IL-6 can inhibit neutrophil apoptosis and prolong their lifespan through multiple pathways, thereby enabling their persistent accumulation at inflammatory sites.^[62,63]^ Neutrophil apoptosis facilitates phagocytosis without causing an inflammatory response, or local cytotoxic effects, which is very important for the normal resolution of the inflammatory process and control of tissue damage.^[63]^

Due to the limitations of the current research conditions and study design, we did not conduct targeted supplementary statistical analyses. Consequently, it is difficult to further quantify the causal relationship between elevated neutrophils and disease severity/inflammatory response using our own clinical data. At present, we cannot directly clarify their specific functional roles. We can only reasonably discuss and speculate on these 2 possibilities and their underlying mechanisms based on relevant domestic and international literature.

In summary, neutrophils and IL-6 play a core regulatory role in the pathogenesis of severe acute urticaria. They interact through a positive feedback loop and jointly promote the exacerbation of inflammatory responses. The positive correlation between neutrophil count and disease severity is essentially an external manifestation of their bidirectional regulation with IL-6 and their own pathogenic effects. Although this speculation lacks direct support from supplementary statistical data in the present study, it is consistent with the findings of existing relevant studies at home and abroad.

Our study had some limitations. Firstly, the sample size was relatively small; the relevant clinical data of this study were mainly derived from a retrospective analysis of 96 patients. Insufficient sample size may introduce bias into statistical results, failing to fully reflect the associations among different populations, various types of infections, and the correlation between neutrophils and the severity of severe acute urticaria, thereby reducing the representativeness of the study findings. Secondly, this study adopted a retrospective design based on archived medical records, which may lead to incomplete data acquisition and lack of regular follow-up. Additionally, the discontinuation criteria for intravenous glucocorticoids were summarized retrospectively from existing clinical documents, rather than prospectively formulated and documented in real time during treatment, potentially causing assessment bias. Finally, prospective intervention on the study subjects cannot be performed, making it difficult to clarify the causal relationships among infection, elevated neutrophils, and the severity of severe acute urticaria, with only their correlations being demonstrable. Finally, the definition of infection was not unified. The diagnosis of infection-related acute urticaria in this study did not adopt a standardized infection determination criterion, and there was a lack of clear specifications for the classification of bacterial and viral infections as well as the grading of infection severity. Differences in the definition of infection across studies may lead to deviations in the degree of association between infection and severe acute urticaria, thereby affecting the consistency of the study results. In addition, the definitions of some variables were relatively broad. The model failed to adequately adjust for key confounding factors, such as antibiotic use, specific glucocorticoid dosages, and tapering schedules. Furthermore, no formal testing was conducted to assess the potential multicollinearity between infection status and neutrophil count, which to a certain extent undermined the robustness of the regression model and the reliability of the study results.

In view of the limitations of this study, future research can expand the sample size and conduct multicenter, prospective clinical studies to reduce statistical bias and improve the representativeness of the research findings. It is necessary to standardize study design and simultaneously conduct in-depth exploration of the specific molecular mechanisms underlying neutrophil-mediated inflammatory responses, especially the interactions between neutrophils and immune cells such as mast cells and eosinophils, so as to provide new targets and insights for the precise treatment of severe acute urticaria.

Meanwhile, in clinical practice, monitoring of neutrophil counts and infection status can be strengthened, and individualized treatment plans can be formulated based on patients’ individual conditions to improve the therapeutic efficacy of severe acute urticaria. Finally, future research and exploration of assessment tools for the severity of severe acute urticaria can focus on the problems exposed in this study, integrate existing commonly used skin assessment tools such as the “Dermatology Life Quality Index,” and incorporate various systemic signs (dyspnea, abdominal pain, and diarrhea), vital signs (blood pressure and heart rate), and various laboratory indicators (IL-6, procalcitonin, C-reactive protein, etc) into the assessment system. Through prospective studies with an expanded sample size, a scientific, accurate, and practical assessment tool can be developed to address the limitations of current tools such as the UAS, provide support for the precise assessment and individualized treatment of infection-related acute urticaria, and ultimately improve the clinical diagnosis and treatment level of severe acute urticaria as well as provide safety guarantees for patients.

## 5. Conclusions

The study determined that infection and elevated neutrophil counts were key factors contributing to the extended duration of intravenous glucocorticoid treatment in patients with severe acute urticaria. The levels of neutrophils, lymphocytes, and WBCs did not indicate whether or not severe acute urticaria was caused by infection. Other laboratory indicators associated with infection-triggered severe acute urticaria require further investigation.

## Acknowledgments

We would like to thank Editage (www.editage.cn) for English language editing. We thank the patients for their written consent to use their data.

## Author contributions

**Data curation:** Mingyi Liao.

**Investigation:** Zhaolin Zeng.

**Methodology:** Shiyin Xiong.

**Supervision:** Zhaolin Zeng, Cong You.

**Validation:** Xinhao Wu, Cong You.

**Writing – original draft:** Kun Wang.
